# Pathology and epidemiology of fatal toxoplasmosis in free-ranging marmosets (*Callithrix* spp.) from the Brazilian atlantic forest

**DOI:** 10.1371/journal.pntd.0010782

**Published:** 2022-09-15

**Authors:** Ayisa Rodrigues Oliveira, Jana M. Ritter, Daniel Oliveira dos Santos, Fabiana Pizzolato de Lucena, Sara Aquino de Mattos, Thaynara Parente de Carvalho, Hannah Bullock, Larissa Giannini Alves Moreira, Izabela Magalhães Arthuso Vasconcelos, Fabíola Barroso Costa, Tatiane Alves da Paixão, Renato Lima Santos

**Affiliations:** 1 Departamento de Clínica e Cirurgia Veterinárias, Escola de Veterinária, Universidade Federal de Minas Gerais, Belo Horizonte, Brazil; 2 Infectious Diseases Pathology Branch, Centers for Disease Control and Prevention, Atlanta, Georgia, United States of America; 3 Setor de Anatomia Patológica, Instituto Municipal de Medicina Veterinária Jorge Vaistman, Rio de Janeiro, Brazil; 4 Synergy America Inc., Duluth, Georgia, United States of America; 5 Departamento de Patologia Geral, Instituto de Ciências Biológicas, Universidade Federal de Minas Gerais, Belo Horizonte, Brazil; Oregon State University College of Veterinary Medicine, UNITED STATES

## Abstract

Toxoplasmosis is an important zoonotic disease that affects a wide range of warm-blooded host species. Neotropical primates (New World Primates; NWP) are highly susceptible, developing a lethal acute systemic disease. Toxoplasmosis in free-ranging NWP is poorly described, with only a few studies based on serosurveys. Herein we performed a retrospective study focusing on the epidemiology and pathology of toxoplasmosis among 1,001 free-ranging marmoset (*Callithrix* spp.) deaths from the Brazilian Atlantic Forest. This study included marmosets necropsied at the Instituto Municipal de Medicina Veterinária Jorge Vaitsman (IJV) from January 2017 to July 2019, which were found dead from all regions in the State of Rio de Janeiro. Histopathology, immunohistochemistry, and transmission electron microscopy were performed to better characterize toxoplasmosis in this free-ranging population. All samples were also tested for Yellow Fever Virus (YFV) RT-qPCR by the official diagnostic service. A total of 1,001 free-ranging marmosets were included in this study, with 16 (1.6%) cases of lethal *Toxoplasma gondii* infections identified both as individual cases and in outbreaks. Presence of infection was not associated with sex, age, geographical distribution, or year of death, and no co-infection with YFV was observed. The main pathological feature in these cases was random necrotizing hepatitis with detection of intralesional *T*. *gondii* zoites in all infected cases. Interstitial pneumonia rich in alveolar foamy macrophages and fibrin deposition, necrotizing myocarditis and necrotizing splenitis were also pathological features in affected marmosets. Therefore, toxoplasmosis was considered the cause of death in 1.6% of free-ranging marmosets in this retrospective series, including some cases associated with outbreaks. Necrotizing random hepatitis was a consistent pathological finding in affected cases and sampling of liver should be ensured from Callitrichid post mortem cases.

## Author summary

Toxoplasmosis is an important zoonotic disease that represents a concern to captive neotropical primates, due to its high susceptibility, with quick lethal course. Large studies of the pathology and epidemiology of toxoplasmosis in in wildlife neotropical primates are rare, rendering it difficult to evaluate the impact of this disease in free-ranging population. This study investigated toxoplasmosis-induced deaths in 1,001 free-ranging marmosets, describing its epidemiology, pathology, and diagnostic tools. Also, this study highlights the importance of histopathology in the differential diagnosis of other important zoonotic diseases that may circulate in that population, such as Yellow Fever.

## Introduction

Toxoplasmosis is a zoonotic disease occurring worldwide, caused by *Toxoplasma gondii*, a protozoan parasite that infects a wide range of warm-blooded animals. Domestic and wild cats are the definitive hosts, in which the sexual form of the coccidia develops, generating infective oocysts that are shed in the feces [1]. Neotropical primates (New World Primates; NWP) are highly susceptible to toxoplasmosis [1–3]. In captive NWP, toxoplasmosis is characterized by a hyperacute to acute, systemic disease with difficult treatment approach, that evolves quickly to death, being sometimes associated with outbreaks with high lethality [1–7]. There are differences in susceptibility between NWP species, with capuchins being considered more resistant than other neotropical species [2,6].

Studies of free-ranging NWP are mainly based on serological analysis using microscopic agglutination test (MAT), with some positive serosurveys in wild populations of marmosets (*Callithrix penicillata*), howler-monkeys (*Alouatta caraya* and *A*. *palliata*), and capuchins (*Sapajus flavius* and *Cebus imitator*) [8–12]. However, due to its acute lethal course, captive NWPs usually die from the infection before developing a humoral response [2], rendering serology of limited usefulness to evaluate the presence of the disease in these animals. In fact, in a study of seroprevalence of toxoplasmosis in a free-ranging population of golden-headed lion tamarins (*Leontopithecus chrysomelas*), which are known to be highly susceptible, none of the animals was serologically positive [8], supporting the notion that serology may not be a reliable tool to assess circulation of *T*. *gondii* in NWP populations.

Furthermore, there are only a few reports of death associated with toxoplasmosis in free-ranging NWP, including three howler monkeys (*A*. *guariba*) [13] and one southern muriqui (*Brachyteles arachnoides*) [5]. All these cases are individual reports, with no characterization of the disease profile in the wild population. Therefore, although well-described in captive animals, the characteristics of this disease in free-ranging NWP warrants further study. To this end, we conducted a three-year-retrospective study focusing on the epidemiology and pathology of toxoplasmosis among 1,001 free-ranging marmosets (*Callithrix* spp.) from the Brazilian Atlantic Forest.

## Material and methods

### Ethics statement

This study was authorized by the government environmental agency (Instituto Chico Mendes de Conservação da Biodiversidade [ICMBio], Brazil) under the SISBIO license number 67014. All procedures strictly adhered to humane care of animals and all applicable laws and regulations, as well as the ethical policies of this Journal.

### Animals

This retrospective study included 1,001 free-ranging marmosets (*Callithrix* spp.) necropsied at the Instituto Municipal de Medicina Veterinária Jorge Vaitsman (IJV, Rio de Janeiro, Brazil) for Yellow Fever (YF) surveillance, during January 2017 to July 2019, that were found dead from all regions in the State of Rio de Janeiro. Animals were previously tested for YF virus (YFV) by quantitative reverse transcription polymerase chain reaction (RT-qPCR) and immunohistochemistry (IHC) by the official Brazilian diagnostic service. Data about geographical origin, sex and age were collected by IJV.

### Necropsy and histopathology

All animals were necropsied by veterinary pathologists at the IJV. Animals were evaluated by gross examination of the carcass and all that were considered suitable for histopathology were included in this study, excluding carcasses with a advanced stage of autolysis. Samples of brain, heart, lungs, liver, spleen, and kidney were fixed in 10% buffered formalin and embedded in paraffin by routine histology. Formalin-fixed paraffin-embedded (FFPE) tissues were sectioned using a microtome (3–4 μm thick) and stained with hematoxylin and eosin (HE) for microscopic evaluation. Other stains, such as Prussian blue, Gram stain and Periodic acid–Schiff (PAS) were performed as needed.

### Immunohistochemistry (IHC)

IHC was performed on all suspected toxoplasmosis cases: cases with intralesional zoites seen by HE stain, or cases with histopathological lesions compatible with *T*. *gondii* infection as previously described [2,6]. FFPE tissue sections were deparaffinized, hydrated, and subjected to antigen retrieval in a pressure cooker at pH 9.0 buffer (Envision). Endogenous peroxidase activity was blocked with 3.5% hydrogen peroxide and to block non-specific protein bindings, slides were incubated in 6% powdered skim milk. A mouse primary monoclonal antibody (IgG2a) targeting p30 membrane of *T*. *gondii* tachyzoite (clone sc-52255; Santa Cruz Biotechnology) was used at a dilution of 1:100. First, slides were incubated in the primary antibody, overnight, at 4°C and then, incubated with an indirect peroxidase polymeric detection kit (Envision) for 30 minutes at room temperature, followed by revelation with 3,3′-Diaminobenzidine (DAB) solution. Slides were counterstained with hematoxylin. Positive controls included sections of liver from a NWP with confirmed *T*. *gondii* infection [2]. Negative controls had the primary antibody replaced by wash buffer.

### Transmission electron microscopy (TEM)

TEM was performed in one case of *T*. *gondii* infection at the Infectious Diseases Pathology Branch at the Centers for Disease Control and Prevention (IDPB-CDC, Atlanta, USA). For TEM, areas of interest from FFPE blocks were selected based on results from HE and IHC. Samples for EM were removed from paraffin blocks using a 1–2 mm biopsy punch, deparaffinized using xylene, rehydrated using a decreasing ethanol series, and post-fixed in 2.5% glutaraldehyde. Samples were then post-fixed with 1% osmium tetroxide, en-bloc stained with 4% uranyl acetate, dehydrated using an increasing ethanol series and acetone, and embedded in Epon-Araldite resin. Samples were then ultrathin sectioned (~50 nm thick), stained with uranyl acetate and lead citrate, and examined on a Tecnai Biotwin electron microscope.

### Statistical analysis

Data were analyzed using the GraphPad Prism software (version 9.0). Descriptive statistics with 95% confidence intervals were used for general analysis. Frequency of histopathological lesions and other variables, such as age, sex, and geographical distribution, were compared between infected and non-infected animals using the Fisher’s exact test. Kappa Coefficient was calculated to analyze the concordance between HE and IHC assays to detect *T*. *gondii* zoites in the tissue.

## Results

### Epidemiology

**Fig 1:**
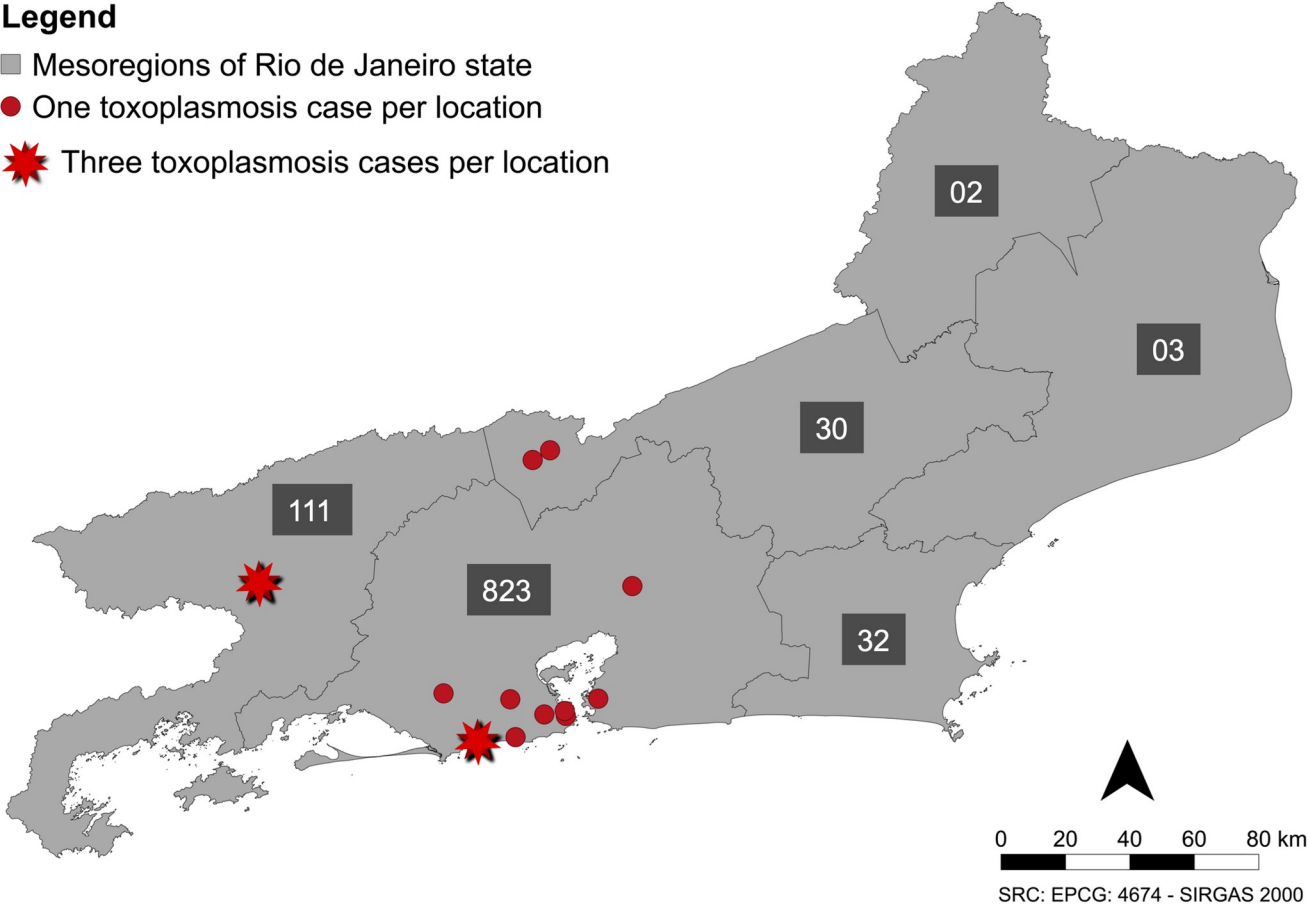
Distribution of toxoplasmosis cases in free-ranging marmosets from Rio de Janeiro state from January 2017 to July 2019. Map from Rio de Janeiro mesoregions (IBGE 2021) with total number of animals with by each mesoregion (grey box). SRC: EPCG 4674 –SIRGAS 2000 (QGIS Version 3.24.3).

A total of 1,001 free-ranging marmoset (*Callithrix* spp.) were included in this study, including 463 females and 463 males (in 75 cases sex was not determined), and 783 adults and 187 juveniles (in 31 cases age was not determined). Toxoplasmosis was diagnosed in 16 animals (1.6%; CI: 1.0–2.6%) (Table 1) from different geographic locations in the State of Rio de Janeiro, Brazil (Fig 1), with 11 cases from the Metropolitan mesoregion (68.7%; 11/16; CI: 44.4–85.8%). In all cases, the animals were found dead in urbanized areas. Eight cases were identified in 2017 (8/304–2.6%; CI: 1.3–5.1%), six in 2018 (6/605–1.0%; CI: 0.4–2.1%), and two in 2019 (2/92–2.2%; CI: 0.4–7.6%). All animals were adults, including 12 females (75%; 12/16; CI: 50.5–89.2%) and four males (25%; 4/16; CI: 10.2–49.5%).

**Table 1:** General data and pathological findings of free-ranging marmosets (*Callithrix* spp.) from the State of Rio de Janeiro (Brazil) with toxoplasmosis from January 2017 to July 2019.

ID	Age	Sex	Date	Origin (city)	Morphologic diagnosis
1	Adult	Female	March, 2017	Rio de Janeiro	Heart: zoites in the cytoplasm of cardiomyocytes, multifocal, moderate. Liver: NH, LHP, random, marked, with intralesional and intracytoplasmic zoites; SPCH, multifocal, moderate, with intraductal trematode. Spleen: splenitis, histiocytic, multifocal, mild, with few intralesional and intracytoplasmic zoites. Kidney: IN, LHP, multifocal, mild, with few intracytoplasmic zoites.
2	Adult	Female	April, 2017	Niterói	Heart: cardiomyocytes necrosis, multifocal, mild, with rare intralesional and intracytoplasmic zoites. Lung: IP, LH, diffuse, mild with moderate multifocal AE and AH, and intralesional and intracytoplasmic zoites. Liver: NH, LHP, random, moderate, with few intralesional intracytoplasmic zoites. Spleen: splenitis, histiocytic, diffuse, moderate, with rare intralesional and intracytoplasmic zoites.
3	Adult	Male	June, 2017	Volta Redonda	Lung: AH, diffuse, moderate; AE, multifocal, mild; moderate amount of alveolar FM with few intralesional intracytoplasmic zoites. Liver: NH, H&N, random, moderate, with intralesional intracytoplasmic zoites and mild random lipidosis. Spleen: splenitis, histiocytic, diffuse, moderate, with intralesional and intracytoplasmic zoites and marked lymphoid hyperplasia. Adrenal: hemorrhage, diffuse, marked.
4	Adult	Female	June, 2017	Volta Redonda	Brain: ME, non-suppurative, multifocal, mild with intralesional intracytoplasmic zoites. Heart: zoites in the cytoplasm of cardiomyocytes, multifocal, rare. Lung: IP, H&N, multifocal, mild with moderate diffuse AE and AH, moderate amount of alveolar fibrin deposition, FM and intralesional and intracytoplasmic zoites. Liver: NH, H&N, random, moderate, with intralesional intracytoplasmic zoites. Spleen: NS, H&N, diffuse, mild, with intralesional and intracytoplasmic zoites and moderate lymphoid hyperplasia. Kidney: IN, LHP, multifocal, moderate, with moderate membranous glomerulopathy and nephrocalcinosis. Adrenal: adrenalitis, LHP&N, multifocal, mild.
5	Adult	Female	June, 2017	Volta Redonda	Heart: NM, multifocal, mild with intralesional intracytoplasmic zoites. Lung: IP, H&N, diffuse, mild with moderate multifocal AE and AH, large number of FM and few intralesional and intracytoplasmic zoites. Liver: NH, H&N, random, moderate, with intralesional intracytoplasmic zoites. Spleen: NS, histiocytic, diffuse, mild, with few intralesional and intracytoplasmic zoites. Adrenal: adrenalitis, LHP&N, multifocal, mild.
6	Adult	Male	July, 2017	Rio de Janeiro	Heart: myocarditis, H&N, multifocal, mild with intralesional intracytoplasmic zoites. Lung: IP, H&N, multifocal, mild with diffuse mild AH, large number of FM and few intralesional and intracytoplasmic zoites. Liver: NH, H&N, random, moderate, with intralesional intracytoplasmic zoites, moderate random lipidosis and moderate SL. Spleen: splenitis, histiocytic, diffuse, mild, with few intralesional and intracytoplasmic zoites.
7	Adult	Male	July, 2017	Rio de Janeiro	Heart: rare zoites in the cytoplasm of cardiomyocytes, multifocal, moderate. Lung: IP, H&N, multifocal, mild with diffuse moderate AE and AH, large number of FM and few intralesional and intracytoplasmic zoites. Liver: NH, H&N, random, moderate, with intralesional intracytoplasmic zoites, moderate random lipidosis and mild SL. Spleen: splenitis, H&N, diffuse, moderate, with few intralesional and intracytoplasmic zoites. Kidney: pigmentary nephrosis, multifocal, moderate, with mild glomerulosclerosis and moderate nephrocalcinosis.
8	Adult	Female	July, 2017	Rio de Janeiro	Heart: myocarditis, H&N, multifocal, moderate with rare intralesional intracytoplasmic zoites. Lung: IP, LH&N, diffuse, moderate with multifocal moderate AE, diffuse marked AH, large number of FM and few intralesional and intracytoplasmic zoites. Liver: NH, H&N, random, moderate, with intralesional intracytoplasmic zoites, marked random lipidosis and mild SL; SPCH, multifocal, moderate, with intraductal trematode. Spleen: NS, H&N, diffuse, mild, with few intralesional and intracytoplasmic zoites. Kidney: IN, LP, multifocal, mild.
9	Adult	Female	February, 2018	Rio de Janeiro	Lung: BIP, H&N, multifocal, moderate with mild multifocal AE and AH, and few intralesional and intracytoplasmic zoites. Liver: NH, H&N, random, moderate, with intralesional intracytoplasmic zoites and moderate random lipidosis. Spleen: splenitis, H&N, diffuse, mild, with few intralesional and intracytoplasmic zoites. Kidney: IN, LP, multifocal, mild. Adrenal: adrenalitis, LHP&N, multifocal, moderate, with multifocal moderate hemorrhage and few intralesional and intracytoplasmic zoites.
10	Adult	Male	March, 2018	Paraíba do Sul	Heart: cardiomyocytes necrosis, multifocal, mild, with rare intralesional and intracytoplasmic zoites. Lung: AE, diffuse, marked and AH, multifocal, moderate. Liver: NH, H&N, random, moderate, with intralesional intracytoplasmic zoites; SCH, multifocal, moderate. Testicle: tubular degeneration, multifocal, moderate.
11	Adult	Female	May, 2018	Rio de Janeiro	Lung: IP, LH&N, diffuse, mild with multifocal mild AH. Liver: NH, H&N, random, moderate, with intralesional intracytoplasmic zoites and moderate random lipidosis; SPCH, multifocal, moderate. Spleen: splenitis, histiocytic, diffuse, mild, with few intralesional and intracytoplasmic zoites. Small intestine: enteritis, histiocytic, diffuse, moderate, with intralesional and intracytoplasmic zoites.
12	Adult	Female	May, 2018	Rio de Janeiro	Brain. Gliosis, multifocal, mild, with rare intralesional and intracytoplasmic zoites. Heart: NM, multifocal, mild with rare intralesional intracytoplasmic zoites. Lung: BIP, H&N, diffuse, mild with diffuse moderate AE. Liver: NH, H&N, random, moderate, with intralesional intracytoplasmic zoites; SPCH, multifocal, moderate, with intraductal trematode. Spleen: splenitis, histiocytic, multifocal, mild, with few intralesional and intracytoplasmic zoites. Kidney: IN, LHP, multifocal, moderate, with membranous glomerulopathy and glomerulosclerosis, multifocal, mild.
13	Adult	Female	July, 2018	Rio de Janeiro	Lung: IP, LH, multifocal, mild with multifocal mild AE and multifocal marked AH. Liver: NH, H&N, random, moderate, with intralesional intracytoplasmic zoites; marked iron storage in hepatocytes. Kidney: IN, LHP, multifocal, moderate, with glomerulosclerosis, multifocal, moderate.
14	Adult	Female	July, 2018	Guapimirim	Heart: myocarditis, multifocal, mild. Lung: IP, LH, diffuse, mild with multifocal mild necrosis, multifocal mild AE, and few intralesional intracytoplasmic zoites. Liver: necrosis, random, moderate with intralesional intracytoplasmic zoites and marked EH. Spleen: Few intralesional intracytoplasmic zoites; lymphoid hyperplasia, mild; EH, mild. Kidney: IN, LP, multifocal, mild. Adrenal: necrotizing adrenalitis, LH, multifocal, moderate, with intralesional intracytoplasmic zoites.
15	Adult	Female	January, 2019	Três Rios	Heart: myocarditis, multifocal, mild. Lung: IP, LH&N, multifocal, mild with multifocal mild AE. Liver: NH, H&N, random, moderate, with rare intralesional intracytoplasmic zoites. Kidney: IN, LP, multifocal, mild.
16	Adult	Female	February, 2019	Campo Grande	Heart: myocarditis, H&N, multifocal, moderate with rare intralesional intracytoplasmic zoites. Lung: IP, LH&N, diffuse, moderate with diffuse marked AE and multifocal mild AH. Liver: NH, H&N, random, moderate, with rare intralesional intracytoplasmic zoites, mild random lipidosis; SPCH, multifocal, moderate, with intraductal trematode. Spleen: splenitis, histiocytic, multifocal, mild, with few intralesional and intracytoplasmic zoites. Kidney: IN, LP, multifocal, mild.

AE (alveolar edema); AH (alveolar hemorrhage); BIP (bronchoIP); EH (extramedullary hematopoiesis); FM (foamy macrophages); H&N (histiocytic and neutrophilic); IN (interstitial nephritis); IP (interstitial pneumonia); LH (lymphohistiocytic); LH&N (lymphohistiocytic and neutrophilic); LHP (lymphohistioplasmacytic); LHP&N (lymphohistioplasmacytic and neutrophilic); LP (lymphoplasmacytic); ME (meningoencephalitis); NH (necrotizing hepatitis); NM (necrotizing myocarditis); NS (necrotizing splenitis); SCH (sclerosing cholangiohepatitis); SL (sinusoidal leukocytosis); SPCH (sclerosing and proliferative cholangiohepatitis)

No statistical difference in the frequency of toxoplasmosis between sex (Fisher’s exact test, p = 0.0743), age (Fisher’s exact test, p = 0.0526), geographical origin (Fisher’s exact test, p = 0.1819) or years (Fisher’s exact test, p > 0.05) was noted. Most incidents were of only one positive individual found dead, however there were two situations (ID3-5 and ID6-8; Table 1) with three positive individuals from the same location, found dead at the same month, suggesting an outbreak in that specific population. Outbreak in this study was defined by multiple cases from the same geographical location found dead in the same month. None of the animals with toxoplasmosis were positive for YFV by RT-PCR at the official diagnostic laboratory.

### Pathological findings

**Fig 2:**
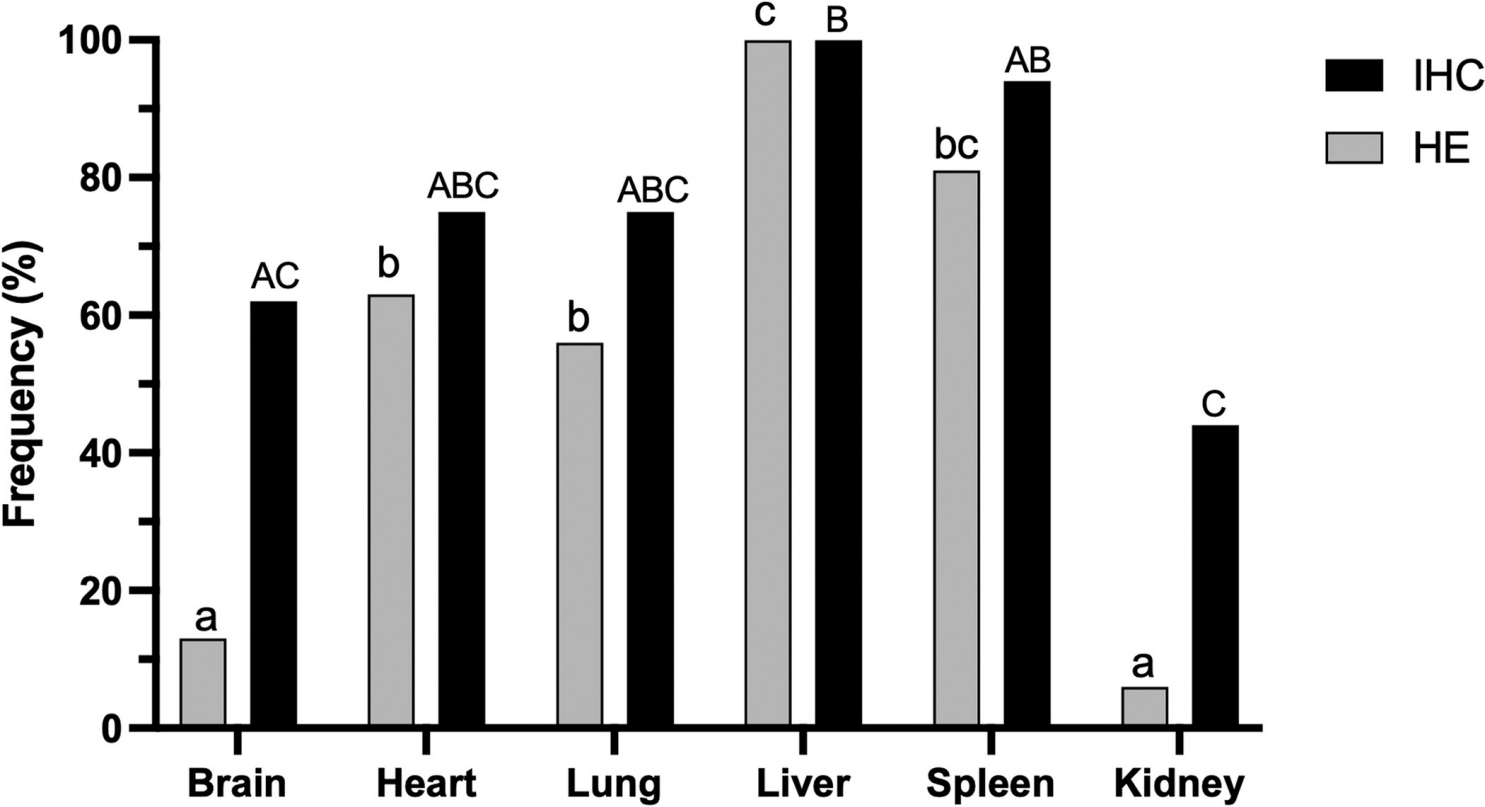
Frequency of intralesional *Toxoplasma gondii* zoites per organ (brain, heart, lung, liver, spleen, and kidney) evaluated by histopathology and immunohistochemistry. Fisher’s exact test was used to compare the distribution frequency of tachyzoites and bradyzoites in the different organs evaluated in HE stained sections (a,b,c: p < 0.05) or by IHC (A,B,C: p < 0.05).

Individual pathological findings are described in Table 1. Based on Kappa statistics, concordance between HE and IHC to detect *T*. *gondii* zoites varied according to the organ analyzed, with almost perfect agreement in the liver (κ = 1), spleen (κ = 0.87) and heart (κ = 0.87), substantial agreement in the lungs (κ = 0.80) and kidney (κ = 0.61), and moderate agreement in the brain (κ = 0.48). Additionally, considering IHC as the gold standard for the diagnosis of toxoplasmosis in FFPE tissue samples, microscopic examination of HE stained sections yielded sensitivities of 100% in the liver, 87% in the spleen, 83% in the heart, 75% in the lung, 20% in the brain, and 14% in the kidney. Frequencies of positivity in different tissues through examination of HE or IHC stained sections are demonstrated in Fig 2.

**Fig 3:**
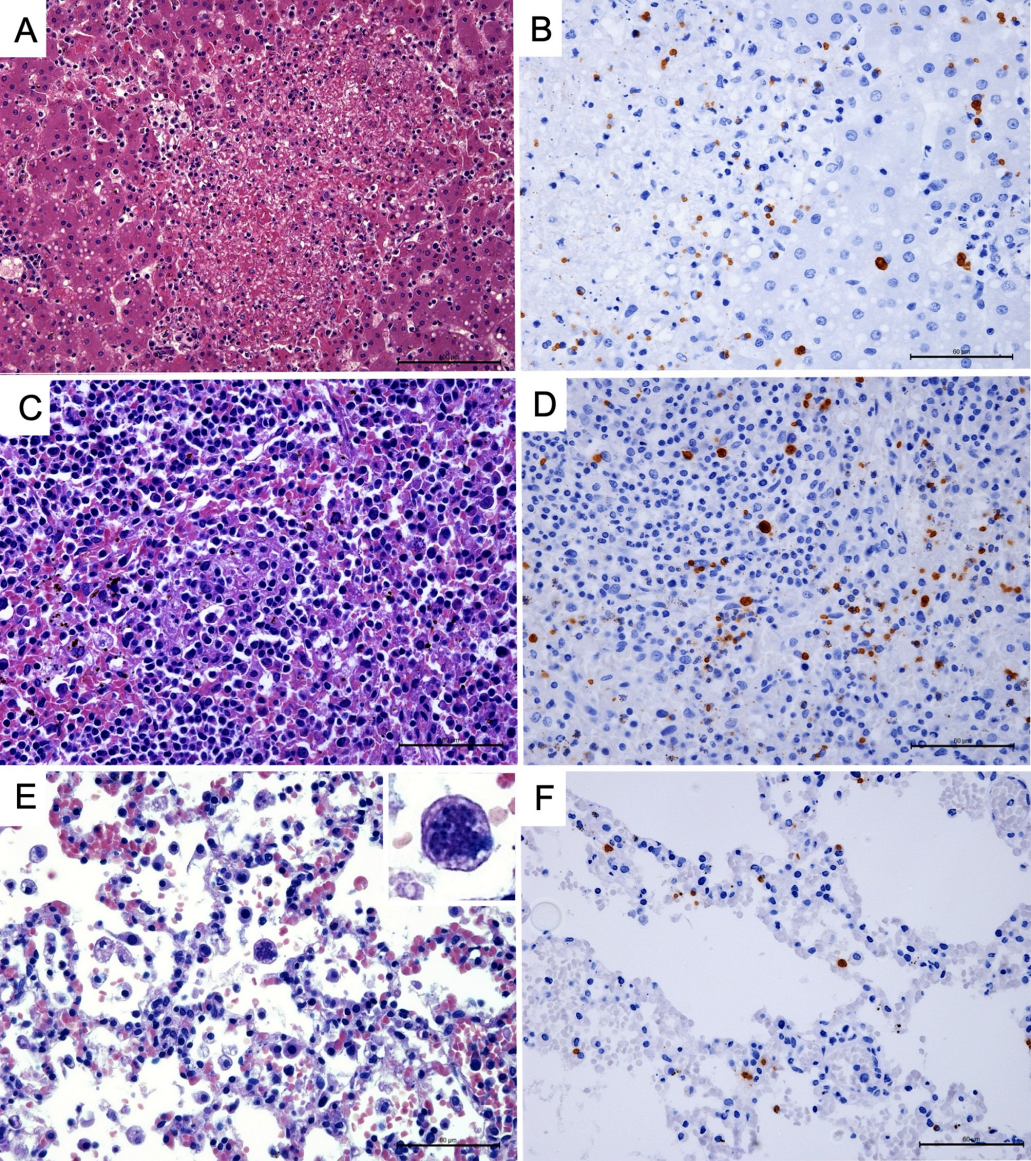
Pathological findings associated with toxoplasmosis in free-ranging marmosets (*Callithrix* spp.). (A) Random lytic necrotizing hepatitis with adjacent mild lipidosis, Liver, HE, Scale bar = 100 μm. (B) Immuno staining for *T*. *gondii* zoites, Liver, DAB, Scale bar = 60 μm. (C) Splenitis, histiocytic, with fibrin deposition in red pulp, Spleen, HE, Scale bar = 60 μm. (D) Immuno staining for *T*. *gondii* zoites, Spleen, DAB, Scale bar = 60 μm. (E) Interstitial pneumonia, characterized by alveolar walls expanded by histiocytes, lymphocytes and plasma cells, with moderate number of foamy macrophages, mild alveolar hemorrhage, diffuse congestion, and intracytoplasmic *Toxoplasma gondii* zoites (inset), Lung, HE, Scale bar = 60 μm. (F) Immuno staining for *T*. *gondii* zoites, Lung, DAB, Scale bar = 60 μm.

**Liver:** Multifocal random lytic hepatocellular necrosis was observed in all infected cases (100%; 16/16; CI: 80.6–100.0%), and together with multifocal random hepatitis (93.8%; 15/16; CI: 71.7–99.7%) and multifocal random lipidosis (43.8%; 7/16; CI: 23.1–66.8%), were the main changes associated with toxoplasmosis in these cases (Fisher’s exact test, p < 0.0001, p < 0.0001, and p = 0.0002, respectively). In most of the cases there was a mild to marked random necrotizing hepatitis with mild to marked multifocal lipidosis, usually observed in the hepatocytes adjacent to necrotic areas (Fig 3A). Intralesional tachyzoites and bradyzoites were observed in all cases (100%; 16/16; CI: 80.6–100.0%), both in HE and IHC stained sections, with significantly higher frequency compared to the other organs (Fig 2). Bradyzoites were identified in the cytoplasm of hepatocytes in areas without inflammation or necrosis, and tachyzoites were observed in Kupffer cells, hepatocytes, and free in the necrotic areas (Fig 3B). Iron storage in hepatocytes, evidenced by Prussian blue stain, was observed in 18.8% (3/16; CI: 6.6–43.0%) of the cases, but was not necessarily associated with the *T*. *gondii* infection (Fisher’s exact test, p = 0.0728). Extramedullary hematopoiesis (12.5%; 2/16. CI: 2.2–36.0%), sinusoidal leukocytosis (18.8%; 3/16; CI: 6.6–43.0%) and syncytial hepatocytes (18.8%; 3/16; CI: 6.6–43.0%) were also observed in these cases but were considered non-specific findings (Fisher’s exact test, p = 0.37, p > 0.9999 and p = 0.7299, respectively). Importantly, there was a significantly higher frequency of cholangiohepatitis with intraductal trematodes, compatible with *Platynosomum* spp., in toxoplasmosis cases (25.0%; 4/16) compared to the frequency of *Platynosomum* spp. in non-infected animals (8.6%; 5/985; Fisher’s exact test, p = 0.0441).

**Spleen:** Splenitis was observed in 75.0% (12/16; CI: 50.5–89.8%) of the cases, characterized by mild to moderate inflammatory infiltrate composed mainly of neutrophils and histiocytes, associated with mild necrosis and fibrin deposition in 18.7% (3/16; CI: 6.6–43.0%) of the cases (Fig 3C). Splenitis and necrosis were both features associated with *T*. *gondii* infection (Fisher’s exact test, p < 0.0001 and p = 0.0113, respectively), and in most cases necrosis was accompanied by mild to moderate deposition of fibrin in the red pulp. Intralesional tachyzoites and bradyzoites were observed in 81.3% (13/16; CI: 57.0–93.4%) of the cases in HE stained sections and in 93.7% (15/16; CI: 71.7–99.7%) by IHC, frequently associated with histiocytes or free at the red pulp (Figs 2 and 3D). Lymphoid hyperplasia (31.2%; 5/16; CI: 14.2–55.6%) and extramedullary hematopoiesis (12.5%; 2/16; CI: 2.2–36.0%) was also observed but were not considered specific features of infected animals (Fisher’s exact test, p = 0.4326 and p > 0.9999, respectively).

**Heart:** Myocarditis and myocardial necrosis were observed in 43.8% (7/16; CI: 23.1–66.8%) of the infected animals (Fisher’s exact test, p = 0.0141 and p < 0.0001 for myocarditis or myocardial necrosis, respectively). Myocarditis and myocardial necrosis were not always observed together; five cases of myocarditis had necrosis, two cases of myocarditis did not have necrosis, and two cases had only myocardial necrosis with no significant inflammatory component. Myocarditis was usually characterized by mild to moderate mixed inflammatory infiltrate composed of lymphocytes, plasma cells, histiocytes, and neutrophils. The myocardial necrosis was mild, focal to multifocal, lytic, and in most cases associated with intralesional tachyzoites, observed free or in the cytoplasm of interstitial histiocytes and cardiomyocytes. Bradyzoites were also observed in the cytoplasm of cardiomyocytes, in most of the cases without inflammatory reaction. *T*. *gondii* zoites in the heart tissue were observed in ten cases (62.5%; 10/16; CI: 38.6–81.5%) in HE stained sections and in 12 cases by IHC (75%; 12/16; CI: 50.5–89.8%) (Fig 2).

**Lungs:** Pneumonia (81.2%; 13/16; CI: 57.0–93.4%) with large number of intra-alveolar foamy macrophages (50.0%; 8/16; CI: 28.0–70.0%) and fibrin deposition (31.2%; 5/16; CI: 14.2–55.6%) were the main findings in infected animals (Fisher’s exact test: p = 0.0029, p < 0.0001, and p = 0.0088 for pneumonia, alveolar foamy macrophages, and alveolar fibrin deposition, respectively). Pneumonia was usually interstitial and broncho-interstitial, mild to moderate, in some cases associated with multifocal areas of interstitial necrosis and mild to marked alveolar edema and hemorrhage (Fig 3E). Intralesional tachyzoites and bradyzoites were observed in nine cases (56.3%; 9/16; CI: 36.2–76.9%) in HE stained sections and in 12 cases by IHC (75%; 12/16; CI: 50.5–89.8%) (Fig 2), usually in the cytoplasm of alveolar macrophages and type II pneumocytes (Fig 3E and 3F).

**Kidney:** No specific findings were observed in the kidneys of infected animals. All had mild to moderate interstitial nephritis composed mainly of lymphocytes, plasma cells and histiocytes; however, this was also a frequent lesion in non-infected animals and was considered a non-specific finding (Fisher’s exact test, p > 0.9999). Tachyzoites and bradyzoites were observed in in the cytoplasm of histocytes in one animal (6.3%; 1/16; CI: 0.3–28.3%) in HE stained sections and in seven animals (43.7%; 7/16; CI: 23.1–66.8%) by IHC. Together with brain, kidney was the tissue with the least frequency of intralesional zoites (Fig 2).

**Brain:** Lesions in the brain were rarely observed in these cases. There was one case of mild non-suppurative meningoencephalitis (6.3%; 1/16; CI: 0.3–28.3%) and one case of mild multifocal gliosis (6.3%; 1/16; CI: 0.3–28.3%). Intralesional tachyzoites and bradyzoites were observed in both cases by HE. IHC detected *T*. *gondii* zoites in ten cases (Fig 2), although in eight, they were not associated with any significant pathological findings.

**Other tissues:** Adrenalitis was observed in four animals, associated with intralesional tachyzoites in two cases. There was one case with diffuse histiocytic enteritis with myriad intralesional tachyzoites.

### Transmission electron microscopy (TEM)

**Fig 4:**
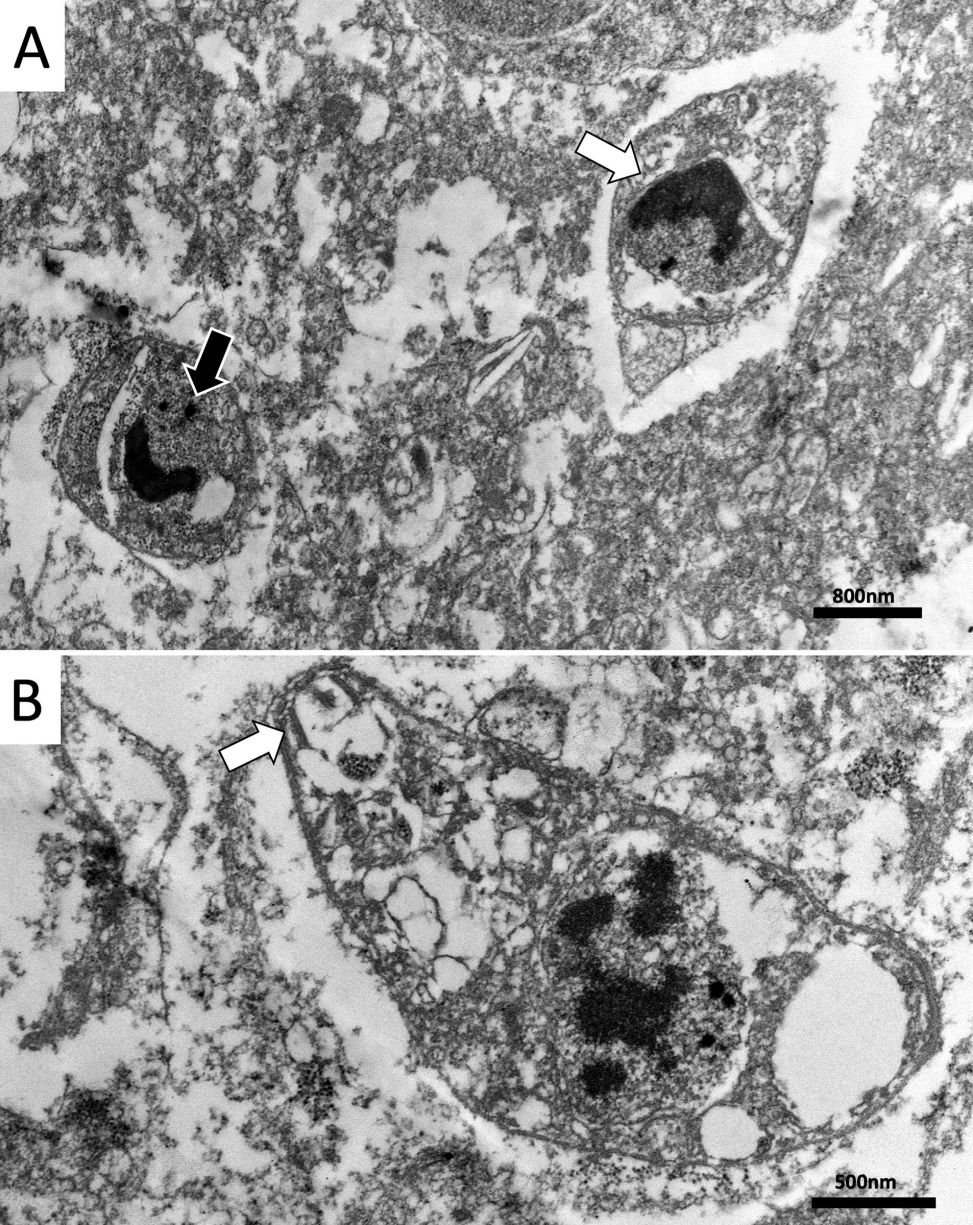
Transmission electron microscopy from the formalin-fixed paraffin-embedded tissue of an infected marmoset. Tachyzoites of *Toxoplasma gondii*, (A) Two ovoid organisms with 1.6 to 2.5 μm x 1.3 to 1.4 μm, a distinct nucleus (white arrow) and electron-dense granules (black arrow), Scale bar = 800 nm. (B) Tachyzoite with one central nucleus and a visible conoid (white arrow), Scale bar = 500 nm.

One of the *T*. *gondii* IHC positive cases (ID14; Table 1) was analyzed by TEM for ultrastructural characterization. Intralesional tachyzoite forms were identified as ovoid-shaped organisms with sizes ranging from 1.6 to 2.5 μm x 1.3 to 1.4 μm and characterized by a round nuclei, dense intracytoplasmic granules, and the presence of a conoid (Fig 4). No bradyzoites were observed.

## Discussion

This study describes the epidemiological and pathological aspects of fatal toxoplasmosis in a free-ranging population of marmosets (*Callithrix* spp.) in Brazil. The prevalence of fatal toxoplasmosis in the population from this study was 1.6%, and was not influenced by sex, age, geographical distribution, or year of death. Fatal toxoplasmosis in free-ranging marmosets was detected as single cases (cases from different geographical regions) and as outbreaks (multiple cases from the same geographical location found dead in the same month) (Fig 1), a similar profile to previously reported toxoplasmosis in captive NWP [2,6,14].

Pathological findings of fatal toxoplasmosis in this study were characterized by a multifocal random necrotizing hepatitis, associated with random lipidosis; interstitial pneumonia rich in alveolar foamy macrophages and fibrin deposition; necrotizing myocarditis; and necrotizing splenitis. These findings are compatible with lesions previously described in cases of NWP lethal infections [1–6,14]. Necrotizing lesions are an important feature in *T*. *gondii* infection and are caused by the direct rupture of the cells by the tachyzoite forms during replication and exit from the host cell, leading to cell death and necrosis surrounded by an acute inflammatory response [15]. *T*. *gondii* tachyzoites also invade and replicate in epithelial respiratory cells, endothelial cells, fibroblasts, and macrophages, causing a disruption in the pulmonary epithelia and capillary barrier, leading to an acute respiratory disease [16]. Lung injuries are frequently described in lethal NWP toxoplasmosis, characterized by acute interstitial pneumonia, in some cases associated with diffuse alveolar damage and detection of alveolar hyaline membranes [2,6,14,17].

After being ingested by an intermediate host, e.g., NWP, the oocysts sporulate in the intestinal lumen by enzymatic degradation and the sporozoites released invade the host cells, changing to tachyzoites that disseminate through blood and lymphatic vessels to most organs [15]. *T*. *gondii* zoites were observed in the liver from all cases of this study (n = 16/16), followed by spleen (n = 15/16), heart (n = 12/16), and lung (n = 12/16), and were less commonly observed in the brain (n = 10/16) and kidney (n = 7/16). These data highlight the importance of liver and spleen in *T*. *gondii* detection, and together with the prevalence of pathological findings, indicate that liver is the organ of choice to be sampled for the diagnosis of toxoplasmosis by histopathology in marmosets (*Callithrix* spp.). Additionally, the concordance between HE and IHC was stronger in the liver, spleen, and heart, indicating that in these tissues, *T*. *gondii* zoites are easily observed, even by standard histopathological evaluation based on HE only. Brain, kidney, and lungs had moderate to substantial agreement, with detection being enhanced by IHC technique.

Random hepatic lipidosis was a frequent feature in toxoplasmosis cases from this study and was also described in previous captive NWP lethal cases [2,6]. In our cases, it was usually observed adjacent to the necrotic areas, being interpreted as a degenerative cellular response to the inflammatory reaction and necrosis caused by the infection. Hemosiderosis has been considered a predisposing factor for toxoplasmosis in NWP [14], being frequently reported in lethal cases [2,14]. However, in this study, hemosiderosis, characterized by iron storage within hepatocytes, was a nonspecific finding, being observed with similar prevalence in infected and non-infected animals.

The increase in density of human population associated with the reduction and fragmentation of wildlife habitats leads to proximity and habitat overlap among humans, domestic animals, and wild primates, dramatically increasing the potential for disease transmission and spillover [18–20]. These inter-species transmission events can directly impact both the survival of wild animal populations and human public health [20]. Marmosets are known for their ability to survive in urban and peri-urban environments, adapting to anthropogenic changes, often consuming human food remains, and exposing themselves to environmental risks of anthropized areas [21]. Importantly, all animals from this report were found dead in urbanized areas, where contact with domestic feline feces is thought to be extremely high, enhancing the exposure of these NWP to *T*. *gondii* infective oocysts. Interestingly, *T*. *gondii* infected marmosets, in our study, had a higher frequency of *Platynosomum* spp. parasitism compared to non-infected animals. Domestic cat is considered the main definitive host in the *Platynosomum* spp. life cycle, as in toxoplasmosis [22]. These data therefore reinforce that marmosets and domestic cats coinhabit the same environments in peri-urban and urbanized areas, and indicate that domestic cats, especially feral and roaming animals, are a potential source of zoonotic pathogens of One Health importance, to both wildlife and humans. Additionally, a serosurvey of toxoplasmosis in asymptomatic free-ranging marmosets from São Paulo, Brazil, found a prevalence of 16.6% [11]. Based on the prevalence of 1.6% of fatal toxoplasmosis observed in our study, we hypothesize that lethality rates in exposed marmoset populations could be approximately 10%, i.e., one of ten marmosets exposed to *T*. *gondii* will probably have a lethal outcome.

Finally, marmosets are sentinels and hosts of other important zoonotic disease with great impact in public health and higher concern in One Health studies, such as YF. During the period of this study the Brazilian Southeast region underwent a major sylvatic YF outbreak, where it is endemic [23,24]. Therefore, YF was an important differential diagnosis in these cases, especially because all these cases coincided with the YF outbreak. Grossly, there are no significant differences between these diseases: both are associated with icterus and an enlargement of the liver that is usually diffusely pale to yellowish, with multifocal reddish areas compatible with hemorrhage [1,2,6,14]. Histopathology is essential to differentiate these two important zoonotic diseases in regions where both occur. YF in susceptible NWP is characterized by a midzonal to massive necrotizing hepatitis with coagulative necrosis, scarce inflammatory cells, and apoptotic hepatocytes, which are traditionally called Councilman-Rocha Lima bodies [23–26]. Animals infected with toxoplasmosis, usually develop a multifocal random necrotizing hepatitis with lytic necrosis, variable inflammatory infiltrate and intralesional zoites, as was observed in our cases [1–6]. Importantly, in agreement with histopathological findings, no infected marmoset from this study was co-infected with YFV, based on negative RT-qPCR and IHC testing for YFV performed at the official diagnostic laboratory.
